# Precipitation behaviour during the β → α/ω phase transformation and its effect on the mechanical performance of a Ti-15Mo-2.7Nb-3Al-0.2Si alloy

**DOI:** 10.1038/s41598-019-54114-0

**Published:** 2019-11-26

**Authors:** Tiewei Xu, Shanshan Zhang, Sen Liang, Ning Cui, Lei Cao, Yong Wan

**Affiliations:** School of Mechanical and Automotive Engineering, Qingdao University of Technology, Qingdao, 266520 China

**Keywords:** Mechanical properties, Metals and alloys

## Abstract

The activation energy of the β → α/ω phase transformation increased monotonously with the application of a continuous heating process to a Ti-15Mo-2.7Nb-3Al-0.2Si alloy. Precipitation behaviour of the alloy aged at different temperatures were analysed with scanning electron microscopy, electron backscattered diffraction and transmission electron microscopy. Selected-area diffraction patterns of the ω, ω/α and α phases in the alloy aged at different temperatures indicated that the type of phase transformation was influenced by the precipitation process. Precipitate-free zones in the alloy aged at 450 °C for 8 h were harmful to the mechanical performance. Fine α precipitates with an obvious texture were obtained in the alloy aged at 500 °C. A good combination of tensile properties with an ultimate tensile strength of 1310 MPa and an elongation of 13.5% were obtained due to the expected microstructure and texture of the precipitates that transformed in the specimen when it was aged at 500 °C for 8 h. The size of the precipitates increased with increasing aging temperature. Furthermore, the amount of precipitates and their degree of texture decreased substantially in the alloy aged at 600 °C. The investigation of the tensile properties and fractures also revealed a correlation between the mechanical properties and precipitation behaviour in the Ti-15Mo-2.7Nb-3Al-0.2Si alloy aged at different temperatures.

## Introduction

Titanium alloys are promising metals for the aviation industry due to their excellent strength and low density^1^. Metastable β-Ti alloys have a single β phase with a bcc crystal structure when their solution treatment temperature exceeds the β/α phase transformation temperature (T_β_)^2,3^. The α and ω phases are both common precipitates which were transformed from the β-phase matrix during aging in titanium alloys. The precipitation of secondary phases (α or ω) formed in a metastable matrix leads to a significant increase in strength^4^. Moreover, the precipitation behaviour in β-Ti alloys is determined by the aging temperature^5^. The β → ω transformation commonly occurs at a low aging temperature due to insufficient thermodynamic energy. The morphology of the precipitates is also influenced by the aging temperature^6^. Angelier^7^ reported mechanisms for the β → α transformation during the aging process of metastable β titanium alloys. The α precipitates could be classified as three types, namely α_GB_, α_WGB_ and α_WM_, according to their nucleation and growth during aging treatment^8,9^.

The evolution of the microstructure and its effect on the performance of beta titanium alloys has been reported in the literature^10–16^. Devaraj *et al*.^17,18^ reported that the ω phase transformed by the collapse of the {111} planes in the matrix structure of a Ti-9Mo alloy. Nag *et al*.^19^ reported that refined α precipitates transformed in the β matrix of Ti-15Mo and TMZF biomedical alloys during 600 °C aging. They also found residual ω phase in a TMZF alloy aged at 600 °C, but it was not found in the Ti-15Mo alloy. Previous researchers aspired to enhance the strength of beta titanium alloys and focused on the relation between the tensile properties and microstructure^14–16,20^. The fine and homogeneous precipitates that occur in a β matrix are expected to improve the thermal stability and mechanical properties of titanium alloys^21–23^. Thus, control of the volume fraction and scale of the precipitates improves the strength and plasticity of beta titanium alloys. Moreover, reasonable methods to improve the strength with an optimal microstructure were expected. Researchers^14,24^ have also studied the relation between the precipitates and mechanical performance of Ti-Mo alloys.

Ti-15Mo-2.7Nb-3Al-0.2Si alloy is a new type of beta titanium alloy that has a complicated phase transformation relationship due to its several alloying elements. The alloy was designed as an alternate material for Ti-6Al-4V to solve the requirements of high strength fasteners in the aerospace industry. This alloy would be optimized to achieve excellent mechanical performance after solution and aging treatments. In the present study, microstructural and textural evolution of a Ti-15Mo-2.7Nb-3Al-0.2Si alloy during aging were investigated systematically by scanning electron microscopy (SEM), transmission electron microscopy (TEM), electron back scattered diffraction (EBSD) and differential scanning calorimeter (DSC) measurements. Furthermore, tensile properties and fractures of the alloy were also studied to reveal the correlation between the mechanical properties and precipitate behaviour in the alloy aged at different temperatures.

## Results

### Activation energy during continuous heating

**Figure 1:**
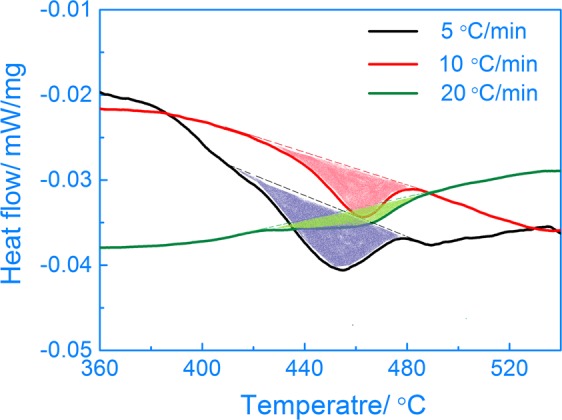
DSC curves of the Ti-15Mo-2.7Nb-3Al-0.2Si alloy heated at 5, 10 and 20 °C/min rates.

Heating rates usually affect the microstructure and performance of Ti-15Mo-2.7Nb-3Al-0.2Si alloys because the progress of the phase transformation is influenced by the thermodynamic condition during the continuous heating process. The activation energy for precipitation is a key kinetic parameter for the β → α phase transformation. The value of the activation energy for phase transformation can be obtained from the results of the endothermic peaks in the DSC measurements performed with different heating rates. Figure 1 shows the DSC curves of the Ti-15Mo-2.7Nb-3Al-0.2Si alloy during continuous heating at 5, 10 and 20 °C/min rates. Heat flow peaks at 410~490 °C indicate that the pivotal phase transformation took place during the continuous heating. Peaks obtained at heating rates of 5~20 °C/min have different shapes due to the variance of the phase transformation in the alloy. The continuous heating at a high rate results in a heat flow peak with a small area. The result indicates that fewer precipitates transformed at a 20 °C/min rate during continuous heating. On the contrary, a large heat flow peak with area at a 5 °C/min heating rate illustrates that an increased number of precipitates were transformed at a low heating rate.

The DSC technique was used to analyse the thermal behaviour of alloys, and kinetic studies have been performed using thermal measurements. According to the Flynn-Wall-Ozawa method, which is commonly used to study the phase transformation of polymorphism, the activation energy of phase transformation in Ti-15Mo-2.7Nb-3Al-0.2Si alloy was calculated, as shown in Eq. 1^25^:

1 $$\mathrm{lg}(\Phi )=\,\mathrm{lg}\,(\Phi A{E}_{f}/R)-\,\mathrm{lg}[{\rm{g}}({f}_{trans})]-2.135-0.4567({E}_{f}/RT)$$

where R, E_*f*_, A and T are the gas constant, the activation energy of the f_trans_, the frequency factor and the absolute temperature, respectively. The value of ϕ is given below for heating at a constant rate from a set temperature. The value of f_trans_ (fraction of phase transformation) is the ratio of the peak area for the heat flow at a temperature point and the whole area of the heat flow peak.

**Figure 2:**
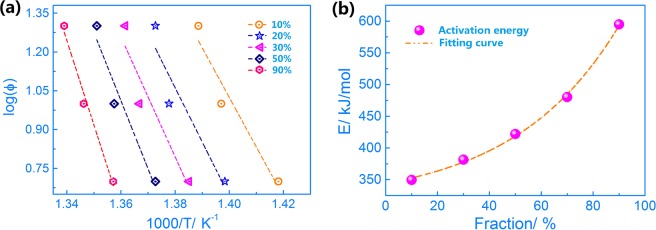
(**a**) Plot of lg (ϕ)-1000/T for the β → α phase transformation in the Ti-15Mo-2.7Nb-3Al-0.2Si alloy; (**b**) Activation energy-Fraction plot for the β → α phase transformation in the Ti-15Mo-2.7Nb-3Al-0.2Si alloy.

Figure 2(a) shows plots for the peaks of the phase transformation in the Ti-15Mo-2.7Nb-3Al-0.2Si alloy during the continuous heating at 5, 10 and 20 °C/min rates. Straight lines were reasonably drawn for each set of data, and activation energies were obtained from the slopes of these lines. The value of lg (ϕ) was plotted vs 1000/T, and the value of lg (*AE*_*f*_ */R*) including the transformation activation energy match due to similar f_trans_ values. Moreover, the transformation activation energy (E_*f*_) under the continuous heating process was calculated using the Flynn-Wall-Ozawa method. Figure 2(b) shows that the activation energy for the β → α phase transformation during heating increases as the phase transformation progresses. The value of the activation energy f_trans_ ≈ 10% is 349.4 kJ/mol. A slight increase in E_*f*_ appeared at the f_trans_ ≈ 10~50% stage of the phase transformation. The values of E_*f*_ are 381.6 and 422.0 kJ/mol for the 30% and 50% phase transformation fractions, respectively, during heating. The value of E_*f*_ is 594.6 kJ/mol when the phase transformation has taken place at the f_trans_ ≈ 90% and is higher than that at the f_trans_ ≈ 70% (480.1 kJ/mol) obviously. The value of E_*f*_ at f_trans_ ≈ 90% increases suddenly because the phase transformation becomes very difficult and additional energy is required at the end of the heating process.

### Nucleation behaviour during the aging process

**Figure 3:**
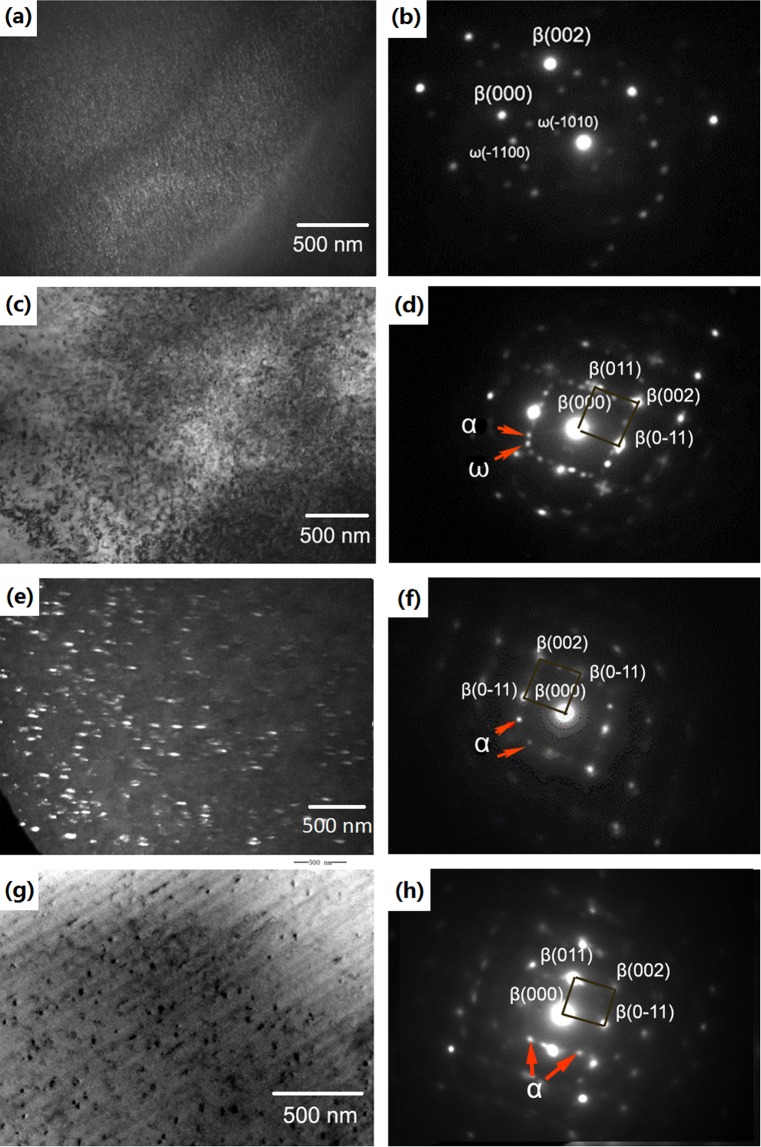
TEM images of precipitates in the Ti-15Mo-2.7Nb-3Al-0.2Si alloy at the beginning stage of the aging treatments: (**a**,**b**) DF image and SAD patterns of the alloy aged at 320 °C for 1 h; **(c**,**d)** BF image and SAD patterns of the alloy aged at 450 °C for 1 h; (**e**,**f)** DF image and SAD patterns of the alloy aged at 500 °C for 0.5 h; and (**g**,**h)** BF image and SAD patterns of the alloy aged at 600 °C for 0.5 h.

Figure 3 shows TEM images and the selected area diffraction (SAD) patterns of the Ti-15Mo-2.7Nb-3Al-0.2Si alloy aged at 320 °C, 450 °C, 500 °C and 600 °C during the beginning stage. The bright-field (BF) and dark-field (DF) TEM images were used to observe the detailed morphology of the precipitates. SAD patterns were also collected to ascertain the crystal structure and habit transformation of the phases during the aging treatments. The DF image (in Fig. 3(a)) illustrates that a large amount of very small spherical precipitates with a size less than 50 nm were transformed during aging at 320 °C for 1 h in the Ti-15Mo-2.7Nb-3Al-0.2Si alloy. The SAD patterns shown in Fig. 3(b) show reciprocal lattice streaking at the 1/3 and 2/3 {112}β positions and indicate that these precipitates were ω phase. These results are consistent with previous reports of [012](200)β//[0-111](1-1-1)ω1//[11-21](01-1)ω2^22,26–28^. The very small precipitates were also observed in the BF image in the alloy aged at 450 °C for 1 h, as shown in Fig. 3(c). The SAD patterns (in Fig. 3(d)) shown as 1/3, 2/3 {011}β and 1/2 {011}β indicate that these precipitates were ω and α phase, respectively. When the alloy was aged at 450 °C, the size of precipitates was 50~200 nm, and the SAD patterns of TEM also indicate that the β → ω/α phase transformation took place simultaneously.

The DF TEM image in Fig. 3(e) shows that homogenous precipitates that appeared in the alloy aged at 500 °C for 0.5 h had a spherical shape and a size of 80 nm. The 1/2 {110}β precipitate patterns indicate that the β/α phase transformation only took place (as Fig. 3(f)). The morphology of the precipitates shows an obvious difference in the Ti-15Mo-2.7Nb-3Al-0.2Si alloy transformed at 600 °C after aging for 0.5 h. Figure 3(g) shows that precipitates formed at 600 °C is fewer than aging at low temperatures. An insufficient nucleation rate is the main feature that occurred during aging at 600 °C due to a decrease in the condensate depression. In this circumstance, the amount of precipitates would decrease sharply even when the β → α transformation was completed after a long aging time. The TEM patterns (in Fig. 3(h)) also indicate that all precipitates that transformed at 600 °C were α phase.

### The α precipitates after different aging treatments

**Figure 4:**
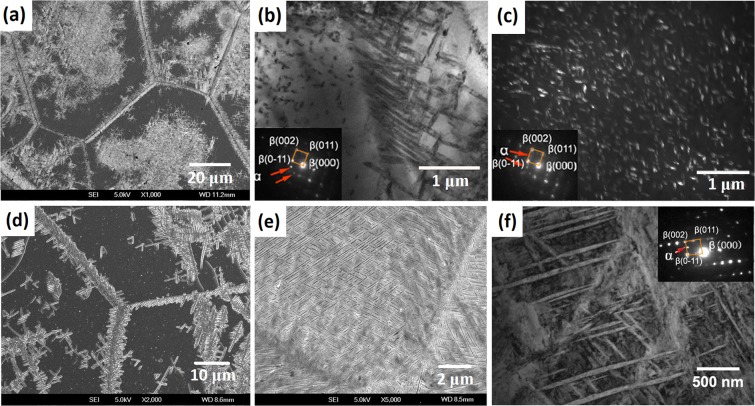
Micrographs of the Ti-15Mo-2.7Nb-3Al-0.2Si alloy after different aging treatments: (**a)** SEM image after aging at 450 °C for 8 h; (**b)** BF image of α_WGB_ precipitates transformed at 450 °C for 8 h; (**c)** DF image of α_WM_ precipitates transformed at 450 °C for 8 h; (**d)** SEM image after aging at 500 °C for 2 h; (**e)** SEM image after aging at 500 °C for 8 h; and (**f**) BF image after aging at 500 °C for 8 h.

Micrographs of the Ti-15Mo-2.7Nb-3Al-0.2Si alloy were also obtained by SEM and TEM to investigate the transformation relation between the α precipitates and β matrix during aging. Figure 4(a–c) show the morphology of the α precipitates in the alloy aged at 450 °C for 8 h. The microstructure shown in Fig. 4(a) includes many α_WGB_ (α Widmanstatten precipitates that developed from the β/β boundaries or α phase at grain boundaries in parallel colonies) and a very small amount of α_WM_ (α Widmanstatten precipitates in the intragranular area). The detailed morphology of the α_WGB_ and α_WM_ precipitates was investigated, as shown in Fig. 4(b,c), respectively. The BF TEM image shows that the α_WGB_ precipitates look like laths with a length of 1 μm. On the other hand, α_WM_ precipitates are spherical, with a size of approximately 80 nm in the DF TEM image. The patterns in the left-bottom corner of Fig. 4(b,c) indicate that the α phase was the sole precipitate phase after 8 h of aging at 450 °C. Moreover, this result suggests that the ω → α transformation completed, per the contrast of the precipitates in Fig. 3(c,d).

The evolution of the precipitates in the Ti-15Mo-2.7Nb-3Al-0.2Si alloy was analysed by comparing the precipitates that transformed during the 2 h and 8 h aging treatments at 500 °C (Fig. 4(d,e)). The SEM micrographs show that the precipitation nucleation at the boundaries and the interior of β grains occurred simultaneously. The length of the lath precipitates transformed at 500 °C was approximately 3 μm after the 2 h and 8 h aging treatments. The difference is that the precipitates fully filled the β matrix grains after 8 h of aging. The patterns in the right-top corner of Fig. 4(f) indicate that all precipitates in the alloy aged at 500 °C were α phase. The BF TEM image shows that α_WM_ precipitates were 80~100 nm wide.

**Figure 5:**
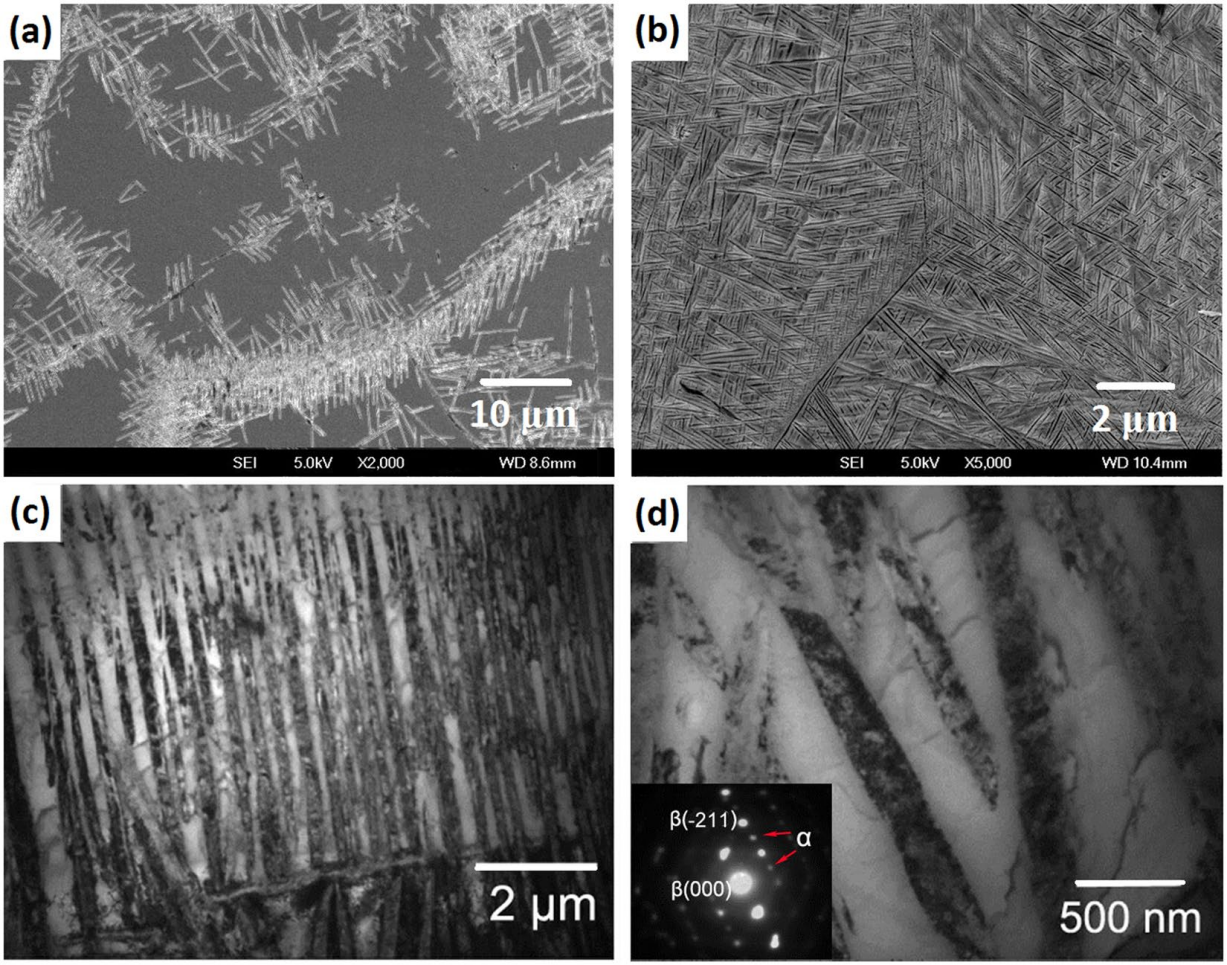
Micrographs of precipitates of the Ti-15Mo-2.7Nb-3Al-0.2Si alloy aged at 600 °C: (**a)** SEM image after aging for 1 h; (**b)** SEM image after aging for 6 h; and (**c**,**d)** BF images of α_WGB_ and α_WM_ precipitates transformed after aging at 600 °C for 6 h.

Similar to the precipitates that transformed at 500 °C, Fig. 5(a) shows that the α_WGB_ and α_WM_ precipitates at the boundary and interior of β grains formed simultaneously in the Ti-15Mo-2.7Nb-3Al-0.2Si alloy aged at 600 °C for 1 h. Furthermore, the length of the lath precipitates is 6~8 μm after this aging condition. The precipitates also fully filled in the β matrix grains after 6 h of aging Fig. 5(b), but the size increases and the amount decreases, in contrast to the precipitation after aging at 500 °C. TEM micrographs of the precipitates at the boundary and in internal grains are shown in Fig. 5(c,d), respectively. The parallel precipitates formed at the boundary and the Widmanstatten precipitates in the intragranular areas have different morphologies. However, the width of the α_WGB_ and α_WM_ precipitates are similar and approximately 200 nm in the alloy aged at 600 °C. The SAD patterns in the left-bottom corner of Fig. 5(d) indicate that the β → α phase transformation occurred solely during 6 h of aging at 600 °C.

### Texture of the alloy aged at different temperatures

**Figure 6:**
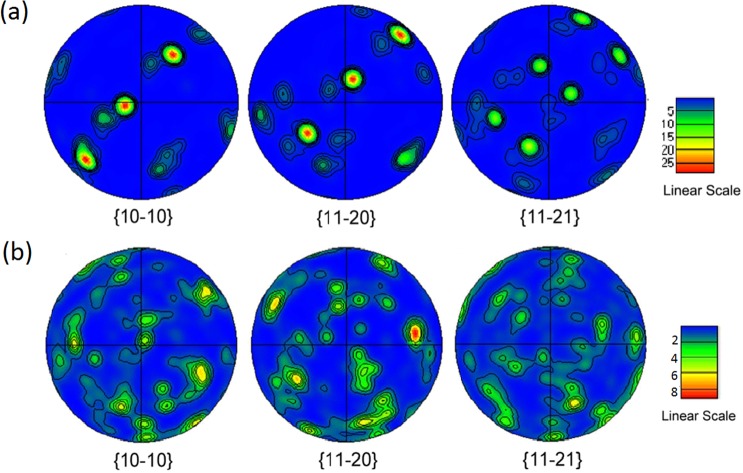
EBSD pole figures of the α phase in the Ti-15Mo-2.7Nb-3Al-0.2Si alloy aged at (**a)** 500 °C for 8 h and **(b)** 600 °C for 6 h. The axial direction is vertical, the hoop direction is horizontal, and the radial direction is approximately the centre.

The EBSD pole figures of the α precipitates transformed during 500 °C and 600 °C aging are shown in Fig. 6(a,b), respectively. The texture of the α precipitates was investigated by (10–10), (11–20) and (11–21) pole figures. The strong {10–10} and {11–20} textures in Fig. 6(a) illustrate that the transformation of β → α in the alloy aged at 500 °C had an obvious texture. In other words, the texture of the alloy and the significant orientation and strengthening of the (10–10) and (11–20) pole figures indicates strong variant selection. After the 500 °C aging process, a definite orientation, which can be observed in the α phase texture, was preserved through the transformation in accordance with the certain texture. Figure 6(a) also shows that the growth of the α precipitates in the 500 °C aged alloy is close to (10–10)α//(011)β, which is related to the Burgers orientation relationship.

The texture of the α precipitates formed during high-temperature aging was also investigated by EBSD pole figures. The temperature that the α precipitates transformed from the β matrix phase in the 600 °C aged alloy is shown in Fig. 6(b). Compared with the pole figures of the specimen aged at 500 °C, the α precipitates in the alloy aged at 600 °C had a significant difference in their crystallographic orientation. There is no obvious variant selection in the 600 °C aged alloy because the projection of {10–10} and {11–20} pole figures is weak and dispersed. Furthermore, the intensity of the texture also illustrates additional attenuated contrast to the alloy aged at 500 °C. The nucleation and growth of the α precipitates must comply with the Burgers orientation relationship. According to previous studies^29–32^, the Burgers orientations with (0001)α//{110}β and <11–20> α// <111> β could provide 12 kinds of relations for β/α transformation. Therefore, it is easy to understand that more selective orientations were developed as the β → α transformation occurred in the same β matrix grain during 600 °C aging. Figure 5(b,c) show that some α-phase clusters were formed in the alloy aged at high temperature and divided the prior β grain into several colonies due to the different texture of each cluster.

### Tensile properties of the aged alloy

**Figure 7:**
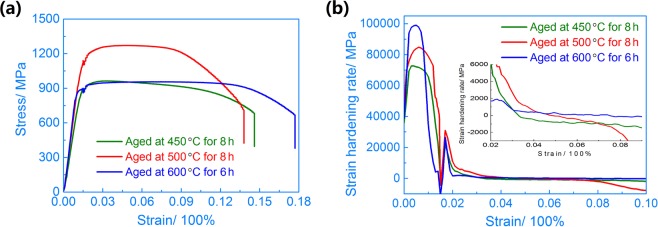
(**a**) Tensile and **(b)** strain hardening rate (SHR) curves of the Ti-15Mo-2.7Nb-3Al-0.2Si alloy aged at 450 °C, 500 °C and 600 °C.

Aging after solution treatment (ST) is expected to form fine α precipitates, which would influence the tensile properties of the beta titanium alloy. Figure 7(a) shows the results of tension testing that was conducted to explore the correlation of the precipitation behaviours and mechanical performance in the Ti-15Mo-2.7Nb-3Al-0.2Si alloy aged at different temperatures. As previously mentioned, the alloys aged at 450 °C, 500 °C and 600 °C had different microstructures and textures (Figs. 3–6). The alloy aged 450 °C for 8 h had an ultimate tensile strength (UTS) of 967 MPa, and the elongation was 14.5% due to large precipitate-free zones (PFZs) in the β matrix phase. The authors found that the tensile properties were strongly influenced by the morphology of the precipitates and residual β phase in previous studies^14,16,24^. The tensile curve of the alloy aged at 500 °C shows that the highest UTS obtained herein was 1310 MPa and the elongation was 13.5%. The precipitates became coarse, and an increased amount of residual β phase existed while the alloy was aged at a higher temperature. Therefore, the value of the elongation increased obviously with increasing aging temperature, and the strength decreased reasonably. The tensile properties of the alloy aged at 600 °C confirmed this trend with an UTS of 942 MPa and elongation of 18%.

Figure 7(b) shows the strain hardening rate (SHR) curves of the alloy aged at 450 °C, 500 °C and 600 °C. Elastic strain was mainly provided by PFZs in the 450 °C aged specimen, due that dislocation pile-ups were formed near α_tiny _precipitates and GBs in the elastic stage. This related strain could be performed by less stress in the PFZs due to few precipitates and low content of solute elements. The SHR curve of the specimen shows a low SHR and indicates there is a low elasticity modulus before yield strength (YS). The central insets of Fig. 7(b) shows the detail SHR curves from 2% to 9% strain in the aged alloy. The SHR of 450 °C aged specimen was a negative value and indicated a softening behavior after YS of tension testing. It implies that the alloy needed less and less stress for plastic strain in the stable strain stage.

A high SHR occurred in the alloy aged at 500 °C during the initial tensile stage, due that many α_WM_ precipitates and GBs hindered dislocation movement and produced pinning effect. And the pinning effect disappeared and crack propagation occurred around GBs and α_WM_ precipitates with increasing tensile strain. The SHR obviously decreased and became a negative value at 5.5% strain (the central insets of Fig. 7(b)). The α_WM_ precipitates showed a lath shape in the alloy aged at 600 °C. These lath-precipitates hindered dislocation movement and formed pile-up groups in the elastic stage. As a matter of course, a slight strain needed a higher stress for broking or bypassing these lath-precipitates. Hence the 600 °C aged specimen has a higher SHR and elasticity modulus in the initial tensile stage. The SHR decreased to near zero after YS and tension testing were in the stable strain stage.

## Discussion

**Figure 8:**
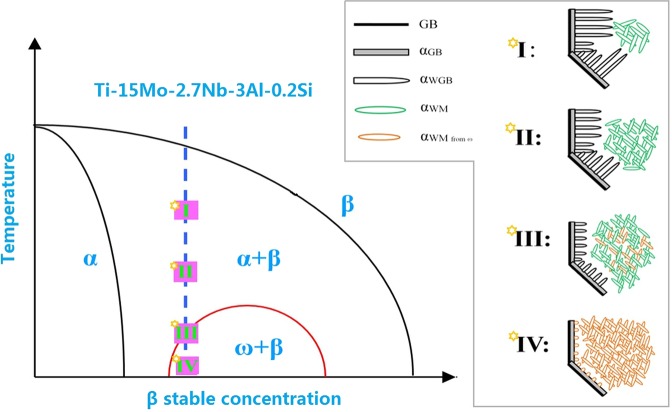
Schematic illustration of phase transformation in the Ti-15Mo-2.7Nb-3Al-0.2Si alloy.

From the results above, a schematic for the transformation in the Ti-3Al-15Mo-3Nb-0.2Si alloy during aging can be summarized, as shown in Fig. 8. The stable β phase concentration in the alloy is 15.75 wt%, which was calculated as the molybdenum equivalent^33^. In the first field, “I” (transformed at an elevated temperature), α_GB_ precipitates nucleated at the grain boundaries and developed into continuous precipitates. Afterwards, the α_WGB_ precipitates nucleated near α_GB_ sites, grew as laths towards the inside of the β matrix and formed a colony structure. Every colony started from a specific boundary facet and developed towards the β matrix grains with a preferred orientation. The phase transformation in the second field “II” (transformed at a high temperature) included α_GB_, α_WGB_ and α_GB_ precipitates simultaneously. The α_GB_ and α_WGB_ precipitates occupied sites near the boundary, but some α_WM_ precipitates nucleated and grew in the interior of the grains. While the aging temperature gradually decreased as the third field “III” (transformed at a moderate temperature), the α_WM_ phase was the main precipitate phase, and the length of the α_WGB_ phase was short. The α_WM_ precipitates appeared in the interior of the grains and simultaneously grew with the α_GB_ and α_WGB_. However, the amount of α_WM_ phase was more in contrast to the precipitation in field “II”. In the fourth field “IV” (transformed at a low temperature), the ω precipitates nucleated in the interior of the grains and provided enough locations for the transformation of the ω → α phase. Phase transformation of the alloy in every field produced a typical microstructure due to the thermodynamic factor of the different aging temperatures. The precipitates mainly consist of the α_GB_ and α_WGB_ phases when the alloy was aged at an elevated temperature. The proportion of α_WM_ precipitates increase with a decrease in the aging temperature, as shown in the illustration (I, II, III and IV) in the right positions of Fig. 8.

**Figure 9:**
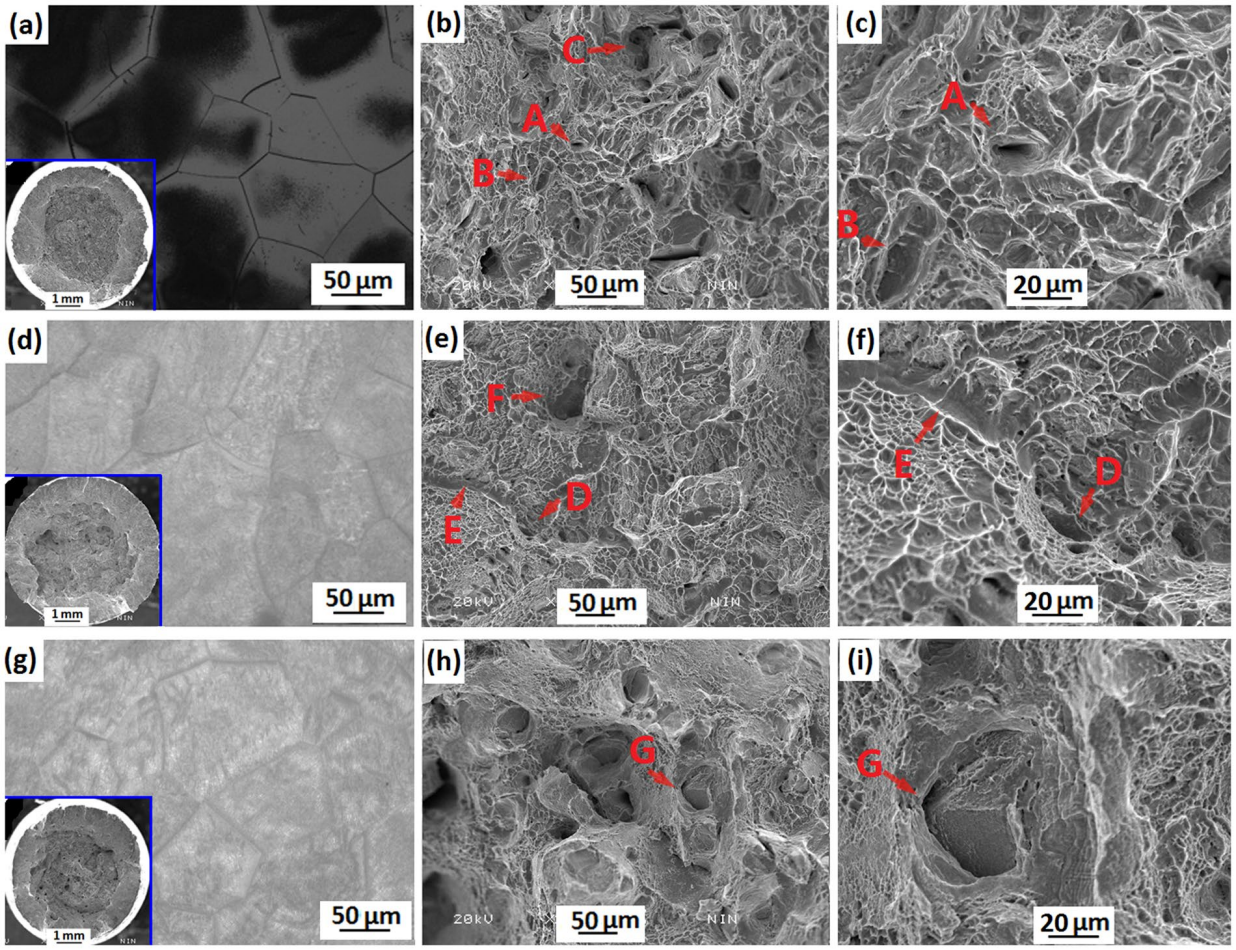
OM micrographs and SEM fractographs of tension testing of the Ti-15Mo-2.7Nb-3Al-0.2Si alloy aged at: (**a**–**c)** 450 °C for 8 h; (**d**–**f**) 500 °C for 8 h; (**g**–**i)** 600 °C for 6 h.

**Figure 10:**
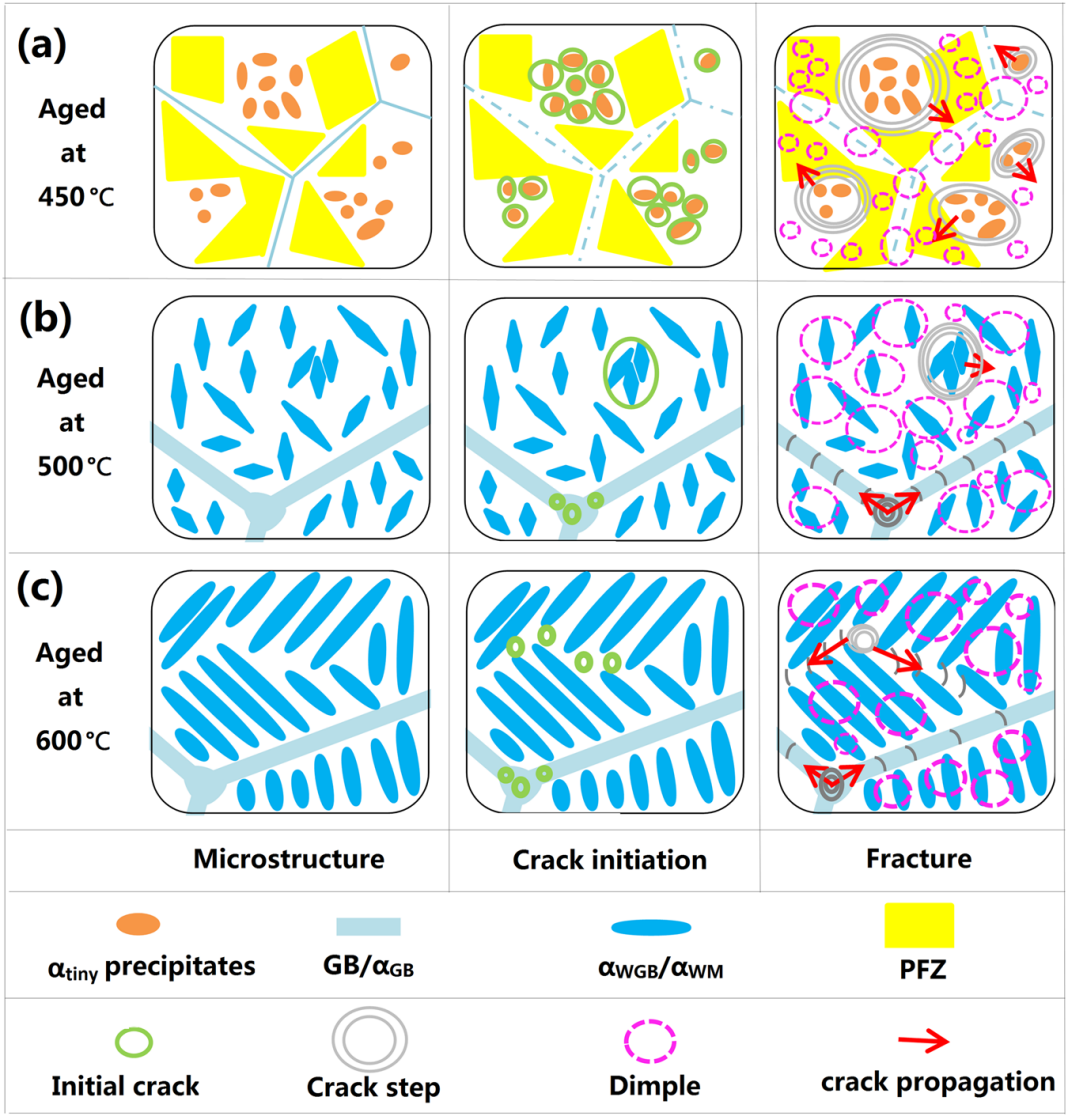
Schematic illustration of the tensile fracture process of the Ti-15Mo-2.7Nb-3Al-0.2Si alloy aged at different conditions: (**a)** 450 °C for 8 h; (**b)** 500 °C for 8 h; (**c)** 600 °C for 6 h.

Figure 9 shows the OM micrographs and tensile fractographs of the Ti-15Mo-2.7Nb-3Al-0.2Si alloy aged at 450 °C, 500 °C and 600 °C after ST plus water-quenching (STQ). The macrographs of the tensile fracture show a difference in the reduction area in the aged specimens (the left bottom of Fig. 9(a,d,g)). The specimen shown in the left bottom of Fig. 9(a), which had a moderate reduction, illustrates that the alloy aged at 450 °C for 8 h had an improved plasticity during tensile deformation. Figure 9(b–d) show that the fracture of the 450 °C aged specimen consisted of many dimples. The microstructure in Fig. 9(a) is considered, these dimples might be deformation traces of the PFZs. Figure 9(a) also illustrates that grain boundaries (GBs) of 450 °C aged specimen are very thin due to less α_GB_ precipitates. These thin GBs hindered dislocation movement and assisted to form dislocation pile-up groups in the initial deformation. However, with increasing tensile strain, these GBs would be broken and formed pinning particles to involve in further deformation. These pinning particles actually were α phase and formed cracks or dimples after final fracture (Fig. 10(a)).

During the initial tensile stage of the alloy aged at 500 °C, α_WM_ precipitates and GBs hindered dislocation movement and produced pinning effect. Micro-holes formed at the position of triangle GBs and particular α_WM_ precipitates when tension testing proceeded near YS (Fig. 10(b)). Sequentially, the pinning effect disappeared and crack propagation occurred around GBs and α_WM_ precipitates with increasing tensile strain (points D and F of Fig. 9(e,f)). The left bottom of Fig. 9(d), with a small reduction, illustrates the alloy aged at 500 °C for 8 h and shows an acceptable tensile plasticity (13.5% elongation, as shown in Fig. 7(a)). However, many very small dimples appeared in the alloy aged at 500 °C, as shown in Fig. 9(e,f). The fine and homogenous α precipitates (as Fig. 9(d)) would be considered for a main reason for the formation of very small dimples at the same depth during tension testing. The high reduction shown in the left bottom of Fig. 9(g) illustrates that the alloy aged at 600 °C for 6 h had the best plasticity during tensile deformation. The α precipitates transformed at 600 °C had various orientations and a large size. Therefore, the fracture was divided into several regions by crack propagation from various depths and circumjacent holes, as shown in Fig. 9(h,i).

Figure 10 show schematic illustration of the tensile fracture process of the Ti-15Mo-2.7Nb-3Al-0.2Si alloy aged at 450 °C, 500 °C and 600 °C. The microstructure of the 450 °C aged specimen is consisted of many α_tiny _precipitates and PFZs. The deformation formed many dislocations pile-up groups and stress concentration around α_tiny_ precipitates while tension testing proceeded near YS. The uncoordinated deformation between α_tiny_  precipitation and β matrix caused crack initiation at the α_tiny_/β interface and formed micro-holes with increasing stress concentration. The SHR of 450 °C aged specimen was a negative value and indicated the softening behavior after YS of tension testing. This phenomenon was due that many cracks had formed at the ω/β interface and the pinning effect disappeared. The stress area is continuously decreased with the increase of fracture holes, and the tensile deformation mainly applied to the PFZs (Fig. 10(a)). This is a main reason why the 450 °C aged specimen needed less and less stress for plastic strain and illustrates a softening behavior in the stable strain stage. Furthermore, these micro-holes expanded into the neighboring β matrix and formed a combined hole during the propagation of cracks (Fig. 10(a)). These combined holes could be observed using SEM and shown as points A, B and C of Fig. 9(b,c). With developing of the tensile deformation, the propagation of cracks occurred in PFZs around the combined holes. To coordinate continual deformation, there are obvious crack-propagation steps formed from the hole to PFZs. Meanwhile, other parts of PFZs supported most of the strain and formed many dimples (Fig. 10(a)).

Microstructure of the specimen aged at 500 °C and 600 °C influenced mechanical properties correspondingly. This influence included many factors such as size, density and texture of the alpha precipitation. Firstly, the α_WM_ precipitates show a short-rod shape in the 500 °C aged specimen and a lath shape in the 600 °C aged specimen. This difference has a great influence on the elastic deformation during tension testing. Precipitation with a short-rod shape could provide enough room for dislocation bypassing α-phase particle, and obtained a low SHR and elasticity modulus in the initial tensile stage. However, lath-precipitates hindered dislocation movement and formed pile-up groups in the elastic stage. This induced that a slight strain needed a higher stress for broking or bypassing of lath-precipitates. Hence the 600 °C aged specimen has a higher SHR and elasticity modulus. Secondly, the density of α_WM_ precipitates was high in the 500 °C aged specimen and low in the 600 °C aged specimen. Conversely, more β matrix was residual in the alloy aged at high temperature and facilitated the plastic deformation to obtain a higher elongation. Thirdly, some α-phase clusters were formed in the 600 °C aged specimen, and divided the prior β grain into several colonies due to the different texture of each cluster (Fig. 10(c)). It is difficult to coordinate deformation between different colonies, and the crack occurred in the joint position of the colonies (point G of Fig. 9(h,i)). However, the α_WM_ precipitates of the 500 °C aged specimen have a similar texture in same prior β grain, and suppress crack initiation in the initial tensile stage (Fig. 10(b)).

## Conclusions

The effects of precipitation behaviour of the Ti-15Mo-2.7Nb-3Al-0.2Si alloy were presented in this study. The microstructure and tensile properties in the alloy aged at different temperatures were investigated by SEM, EBSD, TEM and tension testing. The conclusions are as follows:

1. The activation energy of the phase transformation varied during the continuous heating process, while the alloy was treated at rates of 5, 10 and 20 °C/min. The value of the activation energy was 349.4 kJ/mol at the 10% transformation fraction and had an obvious increase to 594.6 kJ/mol at the 90% transformation fraction.
2. Sole ω precipitation in the alloy aged at 320 °C for 1 h illustrates that the β → α phase transformation was suppressed due to the low aging temperature. However, the transformation of β → α/ω occurred simultaneously in the alloy aged at 450 °C. The β/ω transformation disappeared, and the size of the α precipitates increased with increasing aging temperature. The fine and homogenous α precipitates were obtained in the alloy aged at 500 °C. A decreased amount of lath-like α precipitates transformed during the 600 °C aging process.
3. The α precipitates in the alloy aged at 500 °C had an obvious texture. Orientations of α precipitates in the alloy aged at 600 °C were very dispersed and had a different contrast compared to those transformed at 500 °C. This indicates that the orientation of the transformed precipitates became diversified with increasing aging temperature.
4. Abundant PFZs in the alloy aged at 450 °C for 8 h were detrimental to the mechanical performance. A good combination of tensile properties with an UTS of 1310 MPa and elongation of 13.5% were obtained due to the expected microstructure and texture of the transformed precipitates in the alloy aged at 500 °C for 8 h. The value of elongation increased obviously, and the strength decreased in the alloy aged at 600 °C for 6 h.

## Materials and Methods

The Ti-15Mo-2.7Nb-3Al-0.2Si alloy in this paper was manufactured by Western Superconducting Technology Co., LTD. The alloy was remelted three times using pure Ti, pure Al, SiO_2_, Ti-Nb and Ti-Mo master alloys as raw materials. The chemical composition of the alloy was as follows: Mo = 14.97, Nb = 2.83, Al = 3.01, Si = 0.19, and O = 0.10 mass %. The alloy was hot-rolled to Ф18 mm after homogenizing and forging. STQ was conducted in the β phase region at 840 °C for 1 h and then quenched with the room-temperature water. Continuous heating experiments were performed in STQ specimens with constant rates of 5, 10 and 20 °C/min using DSC measurements. Isothermal aging treatments were performed at 320 °C, 450 °C, 500 °C and 600 °C after STQ. The solution and isothermal aging treatments in this paper were conducted in the ambient atmosphere, and the contaminated surface of the alloy was eliminated in a mechanical manner.

Tension testing was conducted by an INSTRON universal experimental machine at 0.005 mm/mm/min at room temperature. Specimens were prepared for tension testing according to the ASTM E8 standard. The Ti-15Mo-2.7Nb-3Al-0.2Si alloy was etched by 5% HF + 5% HNO_3_ + 90% H_2_O acid (volume fraction) and the metallography was conducted using an OLYMPUSPM-G3 OM. DSC was performed using a TGA/DSC 1/1100 LF analyser operated under vacuum conditions. High-magnification microstructure analysis was conducted using field-emission scanning electron microscopy (FE-SEM) on a JSM6700F instrument. Microstructure analysis was also carried out on a JEM-3010 high-resolution TEM operating at a voltage of 100~300 kV. The TEM specimens were polished to a thickness of 50 μm with 400~2000 grit papers; finally, the twin-jet electrochemical polishing method was used at 243 K (−30 °C) in an electrolyte consisting of perchloric, methanol and butanol (volume ratio 6:35:59). EBSD was performed using a JSM6460 SEM operated at 20 kV. Post-processing of the EBSD data was performed using Channel 5 software from Oxford instruments.

## Data Availability

The datasets generated during and/or analyzed during the current study are available from the corresponding author on reasonable request.
